# Isolated Traumatic Gallbladder Injury: A Rare Case

**DOI:** 10.7759/cureus.43982

**Published:** 2023-08-23

**Authors:** Aashka Shah, Timothy Cho, Faran Bokhari

**Affiliations:** 1 Surgery, Carle Illinois College of Medicine, Urbana, USA; 2 General Surgery, Carle Foundation Hospital, Urbana, USA; 3 Trauma and Burn, Cook County Health, Chicago, USA

**Keywords:** case report, gallbladder wall hematoma, acute cholecystitis, acute traumatic cholecystitis, isolated gallbladder injury

## Abstract

The prevalence of gallbladder injury in a traumatic event is rare, occurring in only 1.9%-2.0% of all abdominal traumas. Isolated gallbladder injuries, without any damage to surrounding organs or tissues, are even less common. Presenting symptoms are often nonspecific, and imaging modalities of ultrasound (US) and CT are usually relied upon to diagnose gallbladder injury accurately. Identifying and strategically treating cases of gallbladder injury, through reviewing this case report, are important for patient outcomes. We present a case of isolated gallbladder injury in a 27-year-old male after sustaining blunt-force abdominal trauma from a snowmobile injury. The patient presented to the emergency department (ED) three days after the initial injury with complaints of significant abdominal pain associated with eating solid food. Upon workup, he was found to have an isolated traumatic gallbladder injury for which a laparoscopic cholecystectomy was performed, and the patient was discharged with no complications. Gallbladder injury, with no evidence of other intra-abdominal injuries, is rare and often not considered in the differential for a trauma patient. Delayed intervention is associated with adverse patient outcomes, emphasizing the need to consider gallbladder injuries in patients presenting with abdominal pain, especially with a history of chronic alcohol use.

## Introduction

Cholecystectomies are one of the most common procedures performed in the operating room, with approximately 300,000 cholecystectomies performed annually [1]. However, the prevalence of gallbladder injury is relatively low in trauma settings due to surrounding parenchymal and rib protection and the small size of the gallbladder [2]. Gallbladder injury is only reported to be about 1.9%-2.1% of all abdominal traumas [3]. Furthermore, an isolated gallbladder injury in a case with blunt abdominal trauma is even rarer. A missed diagnosis of gallbladder injury can cause perforation from an intramural hematoma, which subsequently progresses to necrosis and severe infection [2]. A well-taken history is also imperative in weighing the probability of gallbladder injury, as chronic alcohol use increases its likelihood [4]. The diagnosis of gallbladder injury can be unexpected due to the nonspecific presenting symptoms such as nausea, fever, and right upper quadrant (RUQ) pain, especially in a patient with blunt abdominal trauma, as abdominal pain would be expected due to the mechanism of their injury [5].

In this case, we present a patient who suffered blunt abdominal trauma and presented to the emergency department (ED) three days later with signs of an isolated gallbladder injury.

## Case presentation

A 27-year-old male presented to the emergency department three days after a snowmobile accident complaining of significant right lower quadrant abdominal pain associated with eating solid food. The patient has a history of prior alcohol withdrawal-induced seizures, drinks 12 drinks a week, and has an 11-pack-year smoking history with current smokeless tobacco use. The patient reportedly had been driving 50-60 mph when he was ejected 10 yards forward, causing his body to impact into a nearby tree whereby the primary impact site was the abdomen (specific location unknown). There was no loss of consciousness, and he initially went home after the accident. Upon evaluation in the ED, primary and secondary surveys were notable for moderate right upper quadrant tenderness with guarding resembling localized peritonitis of the right hemiabdomen and otherwise unremarkable with no signs of external injury of the abdomen or the rest of the body. Pulse was slightly tachycardic at 102, and baseline blood pressure was elevated at 147/87 mmHg. He was hemodynamically stable, and laboratory results were notable for a mild leukocytosis to 11.68, a hemoglobin (Hgb) of 10.4, and mildly elevated transaminases (aspartate transaminase {AST} of 127, alanine transaminase {ALT} of 89, and alkaline phosphatase {ALP} of 228). Lipase was within range, decreasing the likelihood of pancreatitis.

Given the mechanism of injury, he was pan-scanned (CT of the brain; cervical, lumbar, and thoracic spine; and abdomen and pelvis {A/P} and right upper quadrant ultrasound {US}). Positive findings on CT of the A/P were consistent with an acute disruption/tear of the gallbladder mucosa, associated gallbladder hematoma/bilioma, and intra-abdominal fluid within the right paracolic gutter and pelvis. After a review of CT of the A/P, radiographic signs were consistent with an isolated gallbladder injury. As such, a RUQ US was ordered for a more focused evaluation of the gallbladder. RUQ US demonstrated gallbladder wall thickening and pericholecystic fluid, possibly due to gallbladder wall hematoma. Cholelithiasis was ruled out. Given these findings and their correlation with recent traumatic injury, it was postulated that he had sustained an isolated blunt traumatic injury to the gallbladder, causing a perforation of the mucosal wall with an associated intramural hematoma/bilioma. The patient was recommended for diagnostic laparoscopy with possible laparoscopic cholecystectomy but decided to opt for an outpatient elective procedure and was discharged on oral antibiotics.

**Figure 1:**
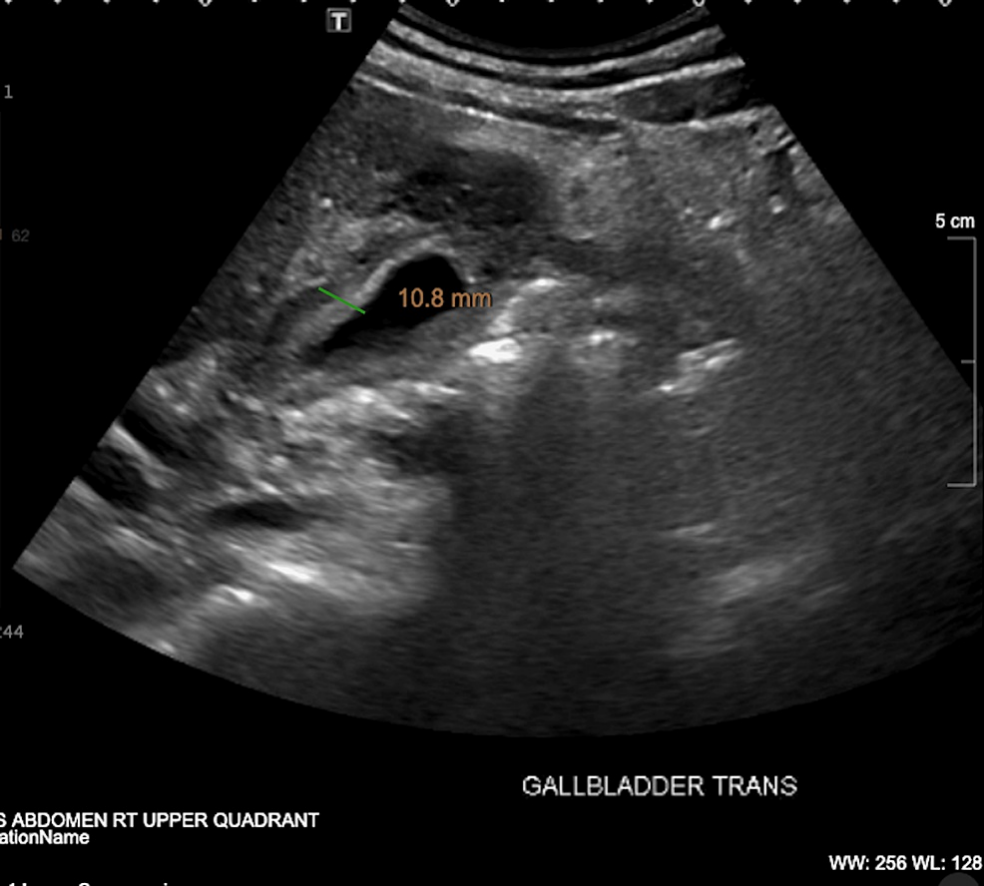
RUQ ultrasound shows diffuse gallbladder wall thickening Gallbladder wall hematoma versus cholecystitis, though less likely RUQ: right upper quadrant

**Figure 2:**
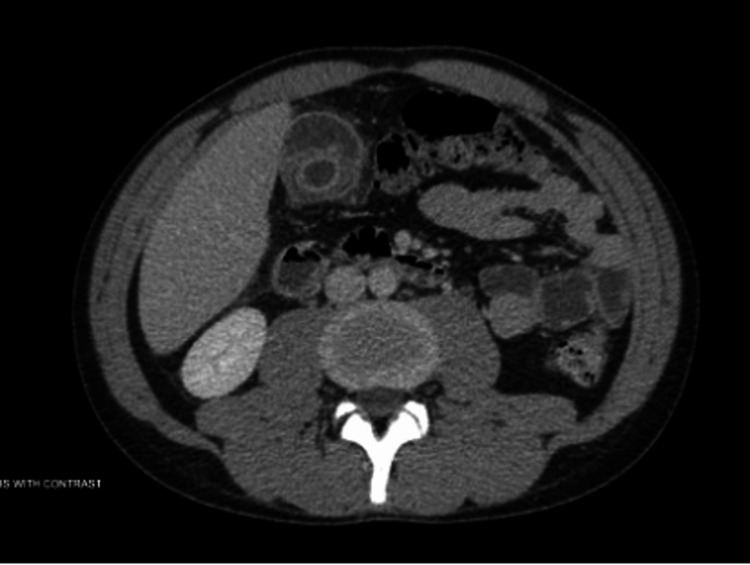
CT of the A/P with persistent heterogenous wall thickening of the gallbladder with intact outer wall and mild increased intraperitoneal fluid Gallbladder distention and adjacent inflammation suspicious for cholecystitis A/P: abdomen and pelvis

**Figure 3:**
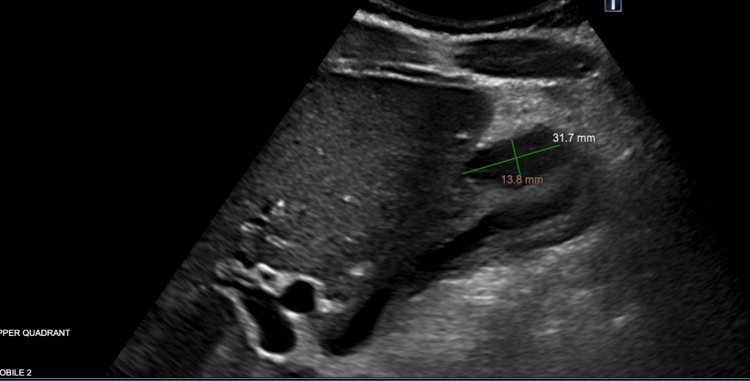
RUQ ultrasound two days later demonstrating possible gallbladder wall hematoma (13.6 mm × 31.7 mm) and wall thickening RUQ: right upper quadrant

Two days later, the patient returned to the ED with worsening recurrent abdominal pain after his son jumped onto his abdomen. A review of systems was negative other than persistent abdominal pain and one episode of emesis. There was continued tenderness to palpation in the right upper and right lower quadrants with voluntary guarding. Laboratory results were remarkable for a resolved leukocytosis (WBC of 7.18) and slightly decreased hemoglobin (Hgb) of 9.8. Liver function tests were also down from the previous ED visit (AST of 107, ALT of 62, and ALP of 230). Repeat CT of the A/P imaging was consistent with prior imaging findings from the initial ED visit of a stable gallbladder mucosal tear with associated gallbladder wall hematoma and mildly increased intraperitoneal fluid non-consistent with blood. Figures 1-3 show the imaging findings throughout admission. Given the recurrence and persistence of his symptoms, he was again recommended for surgical intervention. The patient elected to proceed this time around.

**Figure 4:**
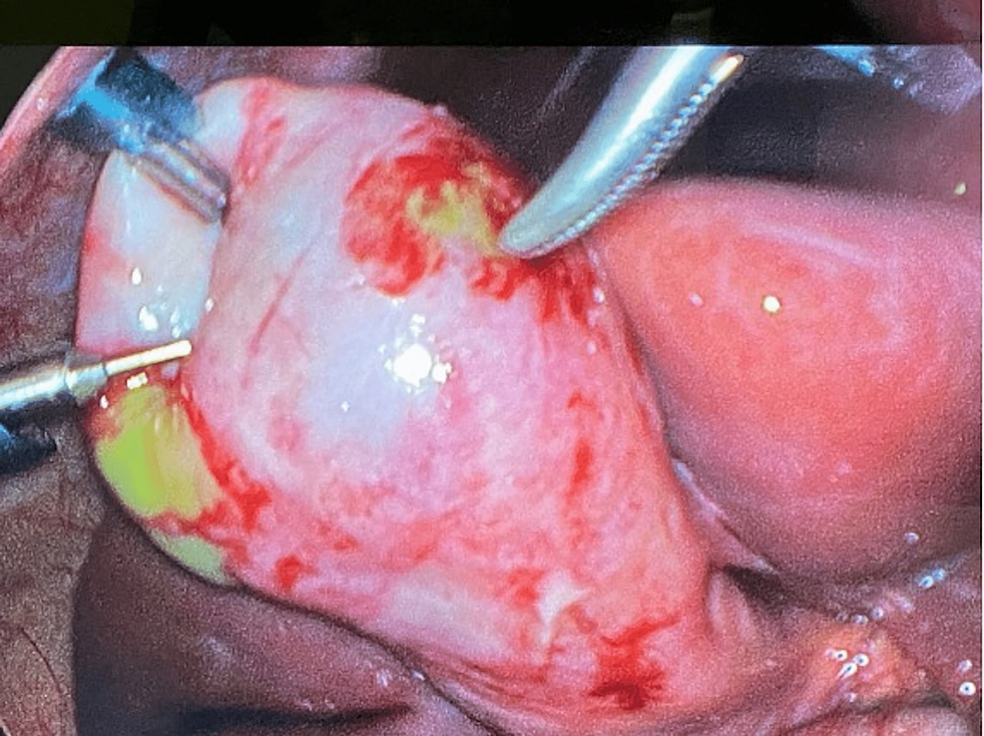
Intraoperative picture of laparoscopic cholecystectomy showing necrosis on gallbladder wall

**Figure 5:**
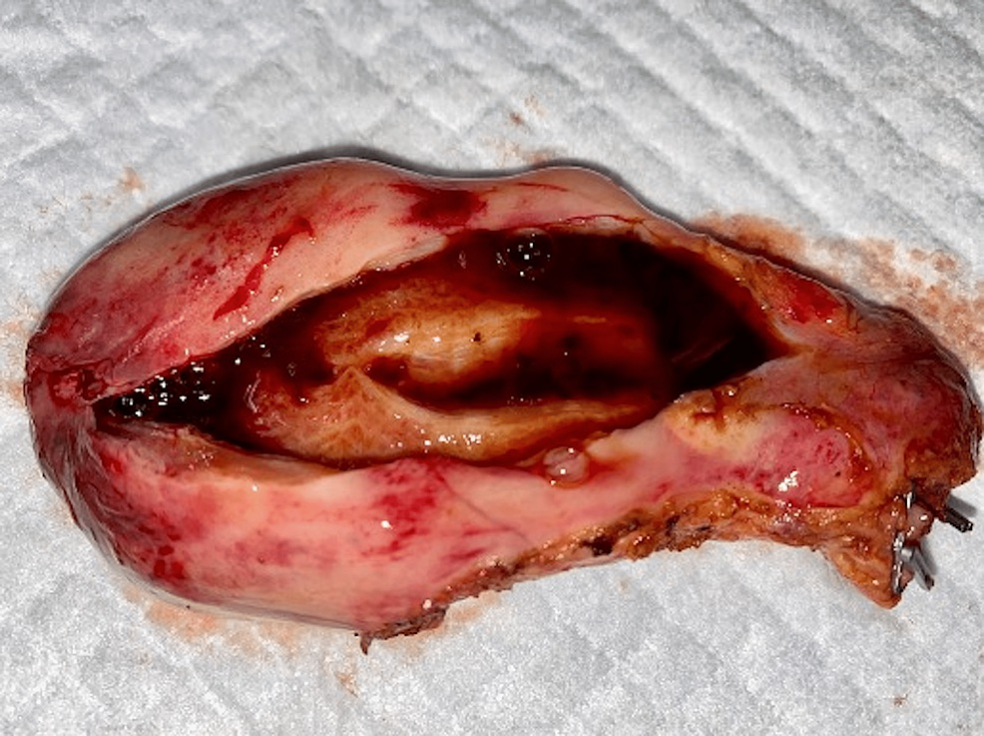
Evidence of gallbladder wall hematoma once removed, on a cut section

Laparoscopic cholecystectomy was performed the following day. The visualization of the gallbladder showed two areas of patchy gangrenous gallbladder wall, and the gallbladder was removed successfully. Intraoperative pictures are seen in Figure 4 and Figure 5. The specimen was sent to pathology, and the pathological reading was acute cholecystitis superimposed on chronic cholecystitis. The patient tolerated the procedure with no post-operative complications. The symptoms resolved, bowel and bladder functions were normal, and the patient was discharged in stable condition on the same day. On a two-week follow-up, he had no post-operative concerns, and his incisions were healing well.

## Discussion

Isolated gallbladder injury is an uncommon result of blunt abdominal trauma. Our patient's initial differential diagnosis included traumatic acute cholecystitis versus acute gallbladder perforation versus intramural hematoma/bilioma. With imaging findings and the nature of his injury, ultrasound findings demonstrated that gallbladder wall hematoma was the most likely. Though cholecystitis was not ruled out, it was less likely. However, CT of the abdomen and pelvis demonstrated gallbladder distension and inflammation suspicious of cholecystitis. The final pathology report confirmed cholecystitis. In this case, ultrasound and CT had two different leading differentials; thus, both were necessary to confirm the diagnosis. Ultrasound of the right upper quadrant is specific and sensitive for acute cholecystitis (81% and 83%, respectively) and therefore should continue to be performed as soon as possible in cases with high suspicion of gallbladder injury as clinical symptoms are nonspecific [6]. CT was also useful in suggesting gallbladder injury as high-density fluid and gallbladder wall thickening can be visualized. This makes ultrasound followed by CT the ideal imaging modality [3,7-9]. In this case, both were necessary for correlating findings. Hepatobiliary scintigraphy can be used if additional imaging is required, as it is the gold standard for diagnosing acute cholecystitis [6]. Provider discretion may be utilized when determining if a hepatobiliary iminodiacetic acid (HIDA) may be warranted.

Blunt gallbladder injuries are classified as contusion, perforation, or avulsion injuries. A contusion injury is an intramural hematoma, while perforation injuries are the most common. Our patient's injuries are most likely consistent with a contained perforation of the gallbladder mucosa. There was no detachment of the gallbladder to suggest an avulsion injury [9]. It is likely that our patient sustained traumatic cholecystitis, which could be related to bile stasis or ischemia, as evidenced by the necrosis of the gallbladder on laparoscopy [10]. With gallbladder injury, concomitant liver (83%-91%), spleen, and duodenum (54%) injuries are highly likely, and special attention to ultrasound should be given to ensure that there are no additional intra-abdominal injuries [9,11]. Fortunately, our patient had no injuries to the surrounding solid or hollow organs.

Patients with gallbladder injuries are 87% male and have a mean age of 27, much like ours [11,12]. Injury to the gallbladder may be more likely in patients who are chronic drinkers as it is probable that the alcohol contributes to gallbladder distension, increasing the likelihood of rupture. Alcohol increases the secretion of bile acids and increases the tone of the sphincter of Oddi. The increased volume and pressure after alcohol consumption can increase the risk of gallbladder injury [13]. Though our patient presented three days after his initial injury and his blood alcohol content at the time of injury was unknown, we postulate that the alcohol could contribute to the traumatic gallbladder injury, given his history. Similarly, most cited cases of gallbladder injury in blunt abdominal trauma had a history of alcohol use, and thus, it is an essential part of the history when considering differentials for patients with a gallbladder injury [2,4,8,11].

This case also highlights the importance of early surgical intervention in the case of acute traumatic cholecystitis. Cholecystectomies performed 1-3 days from injury are associated with better patient outcomes, fewer post-operative complications, and shorter hospital stays [6]. Fortunately, our patient had an uncomplicated operative course; however, given that the procedure was five days after the initial injury, the insistence of earlier surgical intervention would have decreased his risk of potential complications.

## Conclusions

Isolated gallbladder injuries in blunt abdominal trauma are a rare occurrence. There should be high suspicion of these injuries when encountering young adult males with a recent or current history of alcohol abuse. Presenting symptoms may be nonspecific, and imaging, such as CT or US, should be utilized to form an accurate assessment and plan for each patient. There is no current standard of treatment for isolated traumatic gallbladder injuries, but early surgical intervention should be considered to minimize the risk of possible complications and subsequent hospital stays. In this case, the patient felt that his abdominal pain has much improved post-surgery and had a better quality of life.
